# The Effect of Diet Mixing on a Nonselective Herbivore

**DOI:** 10.1371/journal.pone.0158924

**Published:** 2016-07-08

**Authors:** Sophie Groendahl, Patrick Fink

**Affiliations:** University of Cologne, Zoological Institute, Workgroup Aquatic Chemical Ecology, Cologne, NRW, Germany; University of Hyogo, JAPAN

## Abstract

The balanced-diet hypothesis states that a diverse prey community is beneficial to consumers due to resource complementarity among the prey species. Nonselective consumer species cannot differentiate between prey items and are therefore not able to actively regulate their diet intake. We thus wanted to test whether the balanced-diet hypothesis is applicable to nonselective consumers. We conducted a laboratory experiment in which a nonselective model grazer, the freshwater gastropod *Lymnaea stagnalis*, was fed benthic green algae as single species or as a multi-species mixture and quantified the snails’ somatic growth rates and shell lengths over a seven-week period. Gastropods fed the mixed diet were found to exhibit a higher somatic growth rate than the average of the snails fed single prey species. However, growth on the multi-species mixture did not exceed the growth rate obtained on the best single prey species. Similar results were obtained regarding the animals’ shell height increase over time. The mixed diet did not provide the highest growth rate, which confirms our hypothesis. We thus suggest that the balanced-diet hypothesis is less relevant for non-selective generalist consumers, which needs to be considered in estimates of secondary production.

## Introduction

Dietary mixing has been to focus of many studies until today [1–3]. There are two often tested hypotheses which try to explain the advantage of a mixed diet—the balanced-diet hypothesis [4] and the toxin dilution hypothesis [5]. We here focus on the balanced-diet hypothesis, which is the more relevant one for common, non-toxic prey. This hypothesis states that a diverse food resource will result in enhanced consumer fitness. This is due to increased complementarity of the prey species’ nutritional composition. An increase in fitness due to a mixed diet has been observed in a variety of animal groups ranging from ciliates [6], gastropods [7, 8], and insects [9–11], to fish [12], reptiles [13], and mammals [14]. However, in most experiments that investigated the balanced-diet hypothesis, the consumer species were able to freely select what to prey upon. This is often not the case in nature due to costs involved in food search/handling time [15–17], predation risk [18], competition [1], low diversity within the prey communities [19] or defence mechanisms among the prey species [20–22]. These examples demonstrate the many exceptions to the balanced-diet hypothesis. We believe, however, that a very important, but so far overlooked factor is missing on the list. That is the diet selectivity of the consumers. A nonselective consumer cannot choose its prey items and thereby regulate its nutritional intake. It is forced to feed upon the prey in the ratios available, whereas a selective consumer can hand-pick the food items to fit its requirements [23–25]. Moreover, food resources do not have an absolute quality rank. The value of a resource depends on what the consumer has previously been feeding on [26]. Nutrients obtained when feeding upon abundant resources should not be limiting, the preference should therefore always be higher with the rare food items [3]. Nonselective consumers, however, are more likely to consume the more common food resources. If the food recourse of highest availability is of low nutritional value (e.g. cyanobacterial blooms commonly encountered in eutrophied water bodies) this will result in a decreased fitness for the nonselective grazer compared to the selective grazers. However, previous studies were able to demonstrate that selectivity is an advantage if food availability is high [27, 28]. In environments were food availability is low the selective consumer species cannot utilize diet-mixing, it is instead favourable to be a nonselective grazer. Whether the balanced-diet hypothesis is applicable for a nonselective grazer therefore depends on the food quality and availability. Nonselective consumers often have low mobility (*Daphnia*, *Bivalvia* and *Chironomidae*). They are therefore unable to locate food recourses of higher nutritional value. Nonselective consumers are an essential and often dominant component of food webs and they are an important food resource for various animals [29–31]. We thus hypothesize that for nonselective consumers, a mixed diet is not of higher quality than any suitable single diet.

To test this specific hypothesis, we chose to work with the great pond snail *Lymnaea stagnalis* (L.) as a model for a nonselective consumer species. *L*. *stagnalis* has a wide geographic range in the holarctic and it can represent up to 20–60% of the total biomass of macroinvertebrates in many freshwater ecosystems [32]. *L*. *stagnalis* is a scraper feeder which has been shown to be able to detect the quality of biofilms from distance though infochemicals [33, 34]. However, the snails’ feeding apparatus (radula), a minutely toothed chitinous ribbon, is not able to actively select prey organisms from a mixed prey (i.e. biofilm or periphyton) community [35]. However, post-ingestive assimilation may affect the feeding selectivity of *L*. *stagnalis*, but we did not test for this. *L*. *stagnalis* is a suitable model organism since it is not particularly sensitive to dietary changes in nutrient availability [36]. The prey species used in our experimental setup consisted of six pure cultures of benthic green algae *L*. *stagnalis* may encounter in nature. We conducted a laboratory experiment in which we fed juvenile *L*. *stagnalis ad libitum* with either a single algal species or a mixture of the six algal species and determined shell growth over 45 days. We also measured the dry mass of the snails at the beginning and at the end of the experiment to obtain somatic growth rates, as previous studies had demonstrated that juvenile growth rate is a good proxy for fitness in freshwater invertebrates [37]. Overall, our experiment aimed to test the hypothesis that nonselective consumers do not necessarily benefit from diet mixing.

## Material and Methods

Six ten litre bottles each with eight litres of an algal growth medium [38] were inoculated with similar biovolumes of six green algal species (all from the Culture Collection of Algae at the University of Cologne, CCAC, http://www.ccac.uni-koeln.de/, Table 1). All cultures were grown in an environmental chamber at 20°C with a 150 μmol photons s^-1^ m^-2^ light (PAR) intensity under continuous aeration. After 1 month, the batch cultures were harvested by centrifugation and the resulting pellets were freeze-dried. The animals were originally collected in a pond in Appeldorn, NRW Germany, with permission of the owner of the land. The experiment did not involve endangered or protected species. All conditions for animal maintenance and experiments were carefully optimized to meet the animals’ requirements based on extensive prior experience [36]. A specific ethical approval by the university’s IACUC is not required for work with gastropods according to German law. Nevertheless, we undertook all necessary measures to minimize any animal suffering and adhered to the guidelines for the use of animal behaviour for research and teaching (Animal Behaviour 83:301–309). Eggs from adult individuals of the freshwater gastropod *L*. *stagnalis*, were hatched and reared in aquaria filled with aerated tap water. The snails were fed *ad libitum* with (Tetra PlecoMin™) fish food pellets (Tetra, Melle, Germany). The shell height (from the apex to the lower edge of the aperture) was determined to the nearest 0.02 mm using a calliper. A cohort of 64 two-week old *L*. *stagnalis* with a shell height of 2.2 ± 0.3 mm were selected for the experiment. Of these, eight had their shells removed under a dissecting microscope and their soft bodies were dried at 60°C for three days and then weighed with a microbalance (Mettler UTM2, Giessen, Germany) to the nearest microgram to determine the initial dry mass. The remaining 56 snails were subdivided into seven treatments each containing eight replicates. The experiment was conducted in a climatized chamber at 20 ± 0.5°C. The snails were individually placed into square polyethylene containers (length = 11 cm) with 100 ml aged and aerated tap water each. The seven treatments consisted of snails fed with a mixture of all six algae species or one of the six single algal species in saturating and equal quantities.

**Table 1:** Biovolume and cell shapes (d = cell diameter, h = cell height) of the six benthic green algae used in the experiment.

Species	Shape	Measurements (μm)	Standard deviation	Volume (μm3)	Origin/Strain
***Aphanochaete repens***	Sphere	*d* 15	*d* 3.7	1760	CCAC/ M2227
***Klebsormidium flaccidum***	Cylinder	*d* 5, *h* 22	*d* 0.3, *h* 4.9	455	CCAC/2007 B
***Microthamnion kuetzingianum***	90% cylinder with two half spheres, 10% cylinder	*d* 5, *h* 17	*d* 1.2, *h* 4.1	290	CCAC/0087 B
***Oedogonium stellatum***	Cylinder	*d* 12, *h* 40	*d* 1.6, *h* 6.2	4655	CCAC/2231 B
***Roya obtusa***	Cylinder with two half spheres	*d* 8, *h* 48	*d* 1.1, *h* 15.9	2730	CCAC/0219 B
***Stigeoclonium amoenum***	Cylinder	*d* 7, *h* 19	*d* 1.0, *h* 5.0	655	CCAC/3255 B

The snails were transferred into new containers every other day and water and food were renewed on a daily basis. The shell height of the snails was measured in three days intervals. During the course of the experiment, the amount of food provided was gradually increased from 1.5 to 26 mg per individual and day to avoid growth limitation by food quantitybased upon previously estimated ingestion rates (unpublished data). The algae were mixed and then transferred to the snails’ containers through hollow glass cylinders (*d* = 2.3 cm, *h* = 2.5 cm). The glass cylinders were placed in the centre of the containers covered half in water. The algae were then added through the cylinder. After 30 min when the algae had sunk to the bottom of the container the cylinder was carefully removed. This was done in order to avoid the algae from dispersing inside the containers and thereby enabling the snails to selectively feed. After 45 days, the experiment was terminated and the dry mass of the snails was determined as described above.

Juvenile growth increment in *L*. *stagnalis* is assumed to be exponential [39]. Hence, to determine the somatic growth rate [d^-1^] of *L*. *stagnalis*, the following equation was used:

$$g=\frac{\ln{(m}_{end})-\ln\left(m_{start}\right)}{days\;[d]}$$

where the m_start_ is the mean dry mass of the eight snails desiccated at the beginning of the experiment and m_end_ is the dry mass of the snail individual from the respective experimental unit at the end of the experiment (day 45). The relationship between shell length and dry mass of the snails was tested in SigmaPlot (v.11, SysStat) via a nonlinear regression for exponential growth (single, 2 parameter). A one-way ANOVA followed by Tukey’s HSD was conducted in Statistica version 10 to test for significant differences between the somatic growth rates of the snails under the various food regimes. The different food treatments were set as independent variable and the somatic growth rates of the snails were set as dependent variable. Prior to the statistical tests, all data were checked for homoscedasticity using Levene’s test in Statistica. A Mann-Whitney U test was conducted in Statistica to test for a significant difference between the mean somatic growth rates of all single algae treatments and the mixed algal treatment, as the data were not homoscedastic. In order to test for differences in shell heights over time, the data was log transformed and a repeated-measures ANOVA was conducted, followed by *post-hoc* comparisons with Tukey’s HSD in Statistica. Some of the snails did not survive the experiment which is why the number of replicates varied between 6–8 among the treatments.

## Results

**Fig 1:**
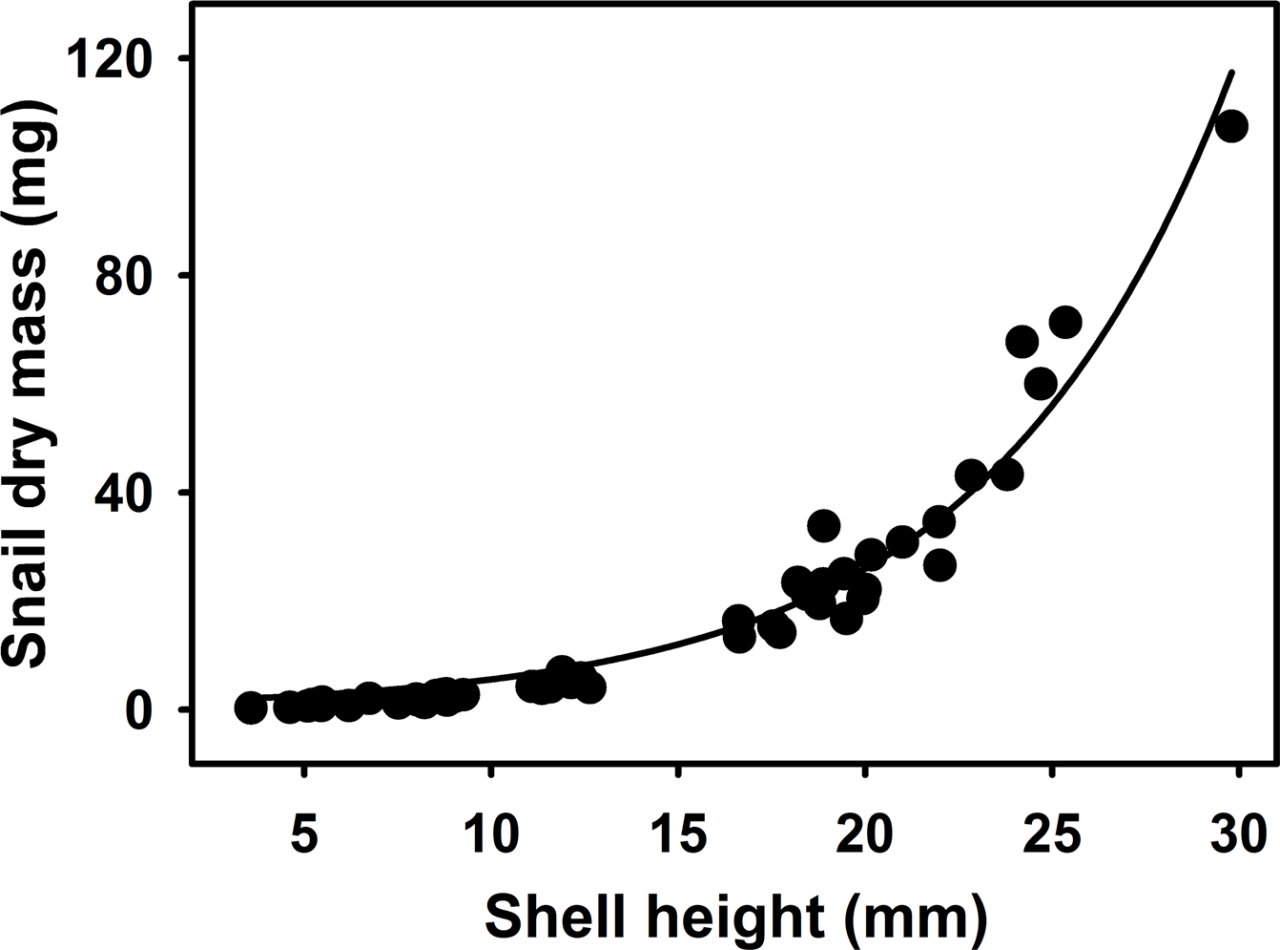
The relationship between shell height and dry mass of *L*. *stagnalis* (exponential regression). Each dot represents one of the 50 snails remaining at the end of the experiment on day 45.

The result from the regression analysis showed that the snails dry mass increased exponentially with the shell height (y = 1.19^(0.15x)^, R^2^ = 0.95, P < 0.0001, Fig 1). The shell heights of the snails varied greatly from 4–30 mm with an average of 14 mm. The dry mass of the snails showed a large variation from 0.5–107.5 mg and an average of 17 mg, suggesting that the different diets did vary considerably in their quality.

**Fig 2:**
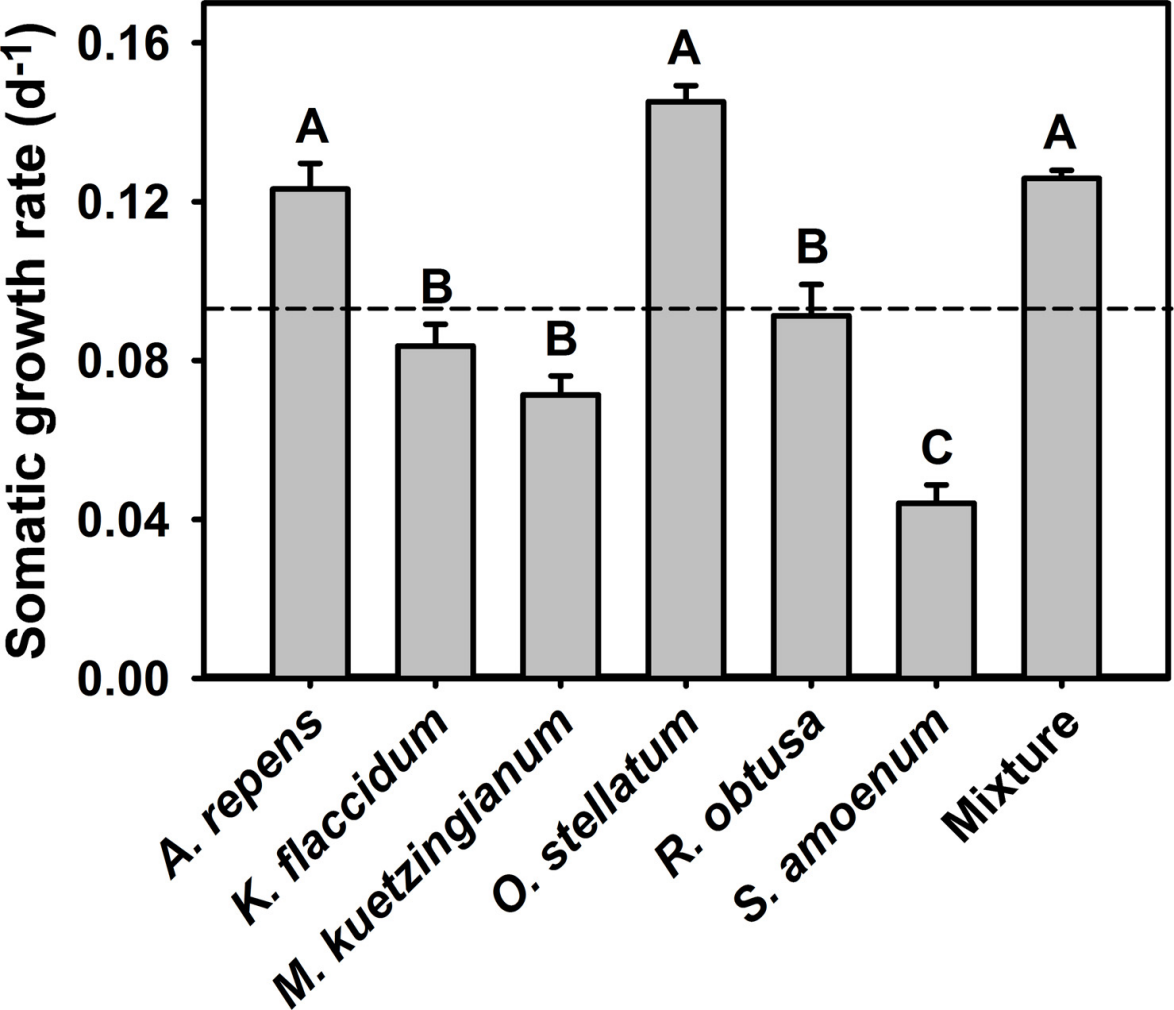
The somatic growth rate of *L*. *stagnalis*. *L*. *stagnalis* fed single algal species or a mixture of all six algal species *ad libitum* where after the somatic growth rates were measured (mean ± SE of N = 6–8).The dashed line indicates the average of all single algal treatments; means which were found to be significantly different after Tukey *post-hoc* comparisons are labelled with different letters.

We found a significant effect of the algae species mixtures consumed on the somatic growth rate of *L*. *stagnalis* (one-way ANOVA, F_6, 51_ = 40.48, P < 0.001, Fig 2). The *post-hoc* comparisons revealed three groups among treatments: One consisting of high growth snails fed the mixed-diet, *A*. *repens* and *O*. *stellatum*, an intermediate growth set of snails fed *K*. *flaccidum*, *M*. *kuetzingianum*, *R*. *obtusa* and the significantly lowest growth was observed in snails fed a diet of *S*. *amoenum* (Fig 2). Juvenile *L*. *stagnalis* fed on the mixed algae exhibited an average (± SE) growth rate of 0.13 ± 0.002 d^-1^, i.e. three times higher than the treatment with the lowest somatic growth rate (Fig 2). In the treatment where snails were fed *O*. *stellatum*, the average (± SE) growth rate was 0.15 ± 0.004 d^-1^ compared to 0.13 ± 0.002 d^-1^ in the mixed treatment. The lowest growth rates were obtained when the snails were fed *S*. *amoenum*. Here, the average (± SE) growth rate was 0.04 ± 0.005 d^-1^. Further, when the snails were fed the mixed algae, the somatic growth rates were significantly higher or equally high as the somatic growth rates of the six single algal species treatments (Mann-Whitney U test, U_1, 56_ = 80, P > 0.02).

**Fig 3:**
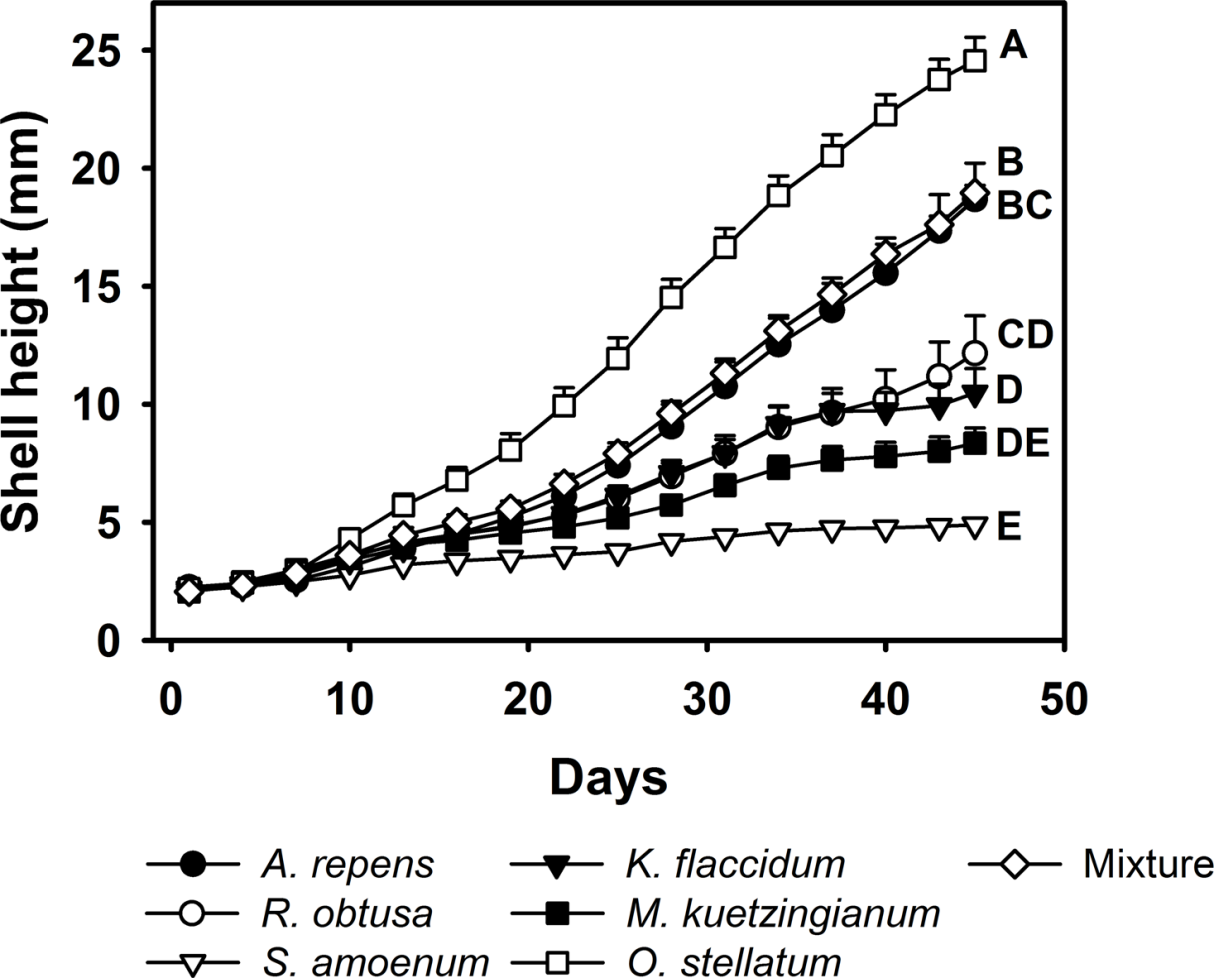
Shell growth of *L*. *stagnalis*. *L*. *stagnalis* fed single algal species or a mixture of all six algal species *ad libitum* during a period of 45 days. Every three days, the shell heights of the experimental snails were measured (mean + SE of N = 6–8); means which were found to be significantly different after Tukey *post-hoc* comparisons are labelled with different letters.

We found significant effects over time between the shell height of the juvenile *L*. *stagnalis* fed with either a single algal species or a mixture of six algal species (repeated measures ANOVA, F_6, 51_ = 30.85, P < 0.0001, Fig 3). Snails fed *O*. *stellatum* had significantly higher shell height increase over time in comparison to all other food treatments (Fig 3). The snails fed with mixed algae exhibited the second largest increases in shell height over time (Fig 3). The shell heights of the juvenile *L*. *stagnalis* were varied greatly between the treatments, at the end of the experiment the snails which fed on *O*. *stellatum* had an average shell height of 24 mm compared to 5 mm when the snails had been fed *S*. *amoenum*.

We further investigated the effect of algae species cell size on snail fitness (shell height). We found that the final shell height increased linearly with the algal biovolume (Table 1) (y = 4.770 + (0.00357x), R^2^ = 0.71, P = 0.035).

## Discussion

We investigated the benefits of a mixed diet compared to a single diet for the growth of a nonselective grazer. The somatic growth rate of *L*. *stagnalis* fed on a mixed diet exceeded the average growth rate for single algal diets. However, the growth of *L*. *stagnalis* fed upon two single species *O*. *stellatum* and *A*. *repens* were not significantly different from growth with the mixed diet. Similar results were obtained regarding the animals’ shell height increase over time. Snails that had consumed *O*. *stellatum* had a higher shell height increase in comparison to all other treatments. The mixed diet thus did not provide the highest growth rate, which confirms our hypothesis that a mixed diet is not more beneficial for nonselective consumers compared to any suitable single diet.

In many studies investigating the effect of diet mixing on consumer species, the prey items were collected from various sites in nature [7, 40] or consisted of artificial food mixtures [14]. This means that the macronutrient ratios within those prey items most probably varied. However, by growing algae in a high nutrient growth medium and harvesting in the exponential growth phase probably led to similar macronutrient contents of the algae in our experiment [41]. Moreover, the fatty acid contents are probably similar among freshwater green algae. The macronutrient content and the fatty acid concentration of the algae therefore probably do not explain the observed differences in growth of the consumer species between the treatments. It is more likely that the algae varied in their ingestibility or digestibility due to morphological defences such as spines, mucilaginous coating and rigid cell walls [21, 22, 42]. We found that the shell height of the snails increased significantly with algae species biovolume. We believe that this can partially explain the observed differences in shell height/growth rates between the treatments. Previous studies were able to demonstrate prey size selection with snails [43, 44]. It has been suggested that a radula can more easily handle larger algal cells.

Consumer species may use various strategies to increase their somatic growth rates when food items are scarce or of low quality. One such strategy is called ‘compensatory feeding’: When consumers feed upon a nutritionally low quality food items they should increase the consumption rate in order to compensate for nutrients which are in low supply [45–47]. An alternative strategy is called ‘toxin dilution’ [5]: Diet mixing reduces (dilutes) the amount of toxins produced by individual prey species that are ingested by the consumer. The toxic dilution hypothesis is not likely to apply since no toxins have been described within the algae species used in the experiment [48, 49]. Further investigations need to be carried out in order to elucidate which explanation, or combination thereof, is correct. We suspect that differences in cell size and compensatory feeding are the major drivers for the results in our experiment.

Lefcheck et al. [50] conducted a meta-analysis in which they investigated the impact of mixed diets on the fitness of animals. They found that in more than 50% of the cases, a single species diet was superior to a mixed diet. However, the impact of the consumer species’ feeding selectivity was not included in the analysis. We argue that this should have strong implications to the result. The grazer used in our experiment could not select their food items in order to optimize their growth. A selective grazer may, which increases the probability of finding support for the balanced-diet hypothesis. We therefore argue that the generality of the balanced-diet hypothesis might be overestimated considering nonselective grazers. Very few studies investigate the impact of the balanced-diet hypothesis on nonselective grazers [51], even less were able to find support for it [52]. However, a few studies worked with species able to conduct both selective and nonselective feeding and examined which feeding strategy a consumer species decides for under various conditions. Senior et al. [53] and Khait et al. [54] found that nonselective grazers should be favoured under low food availability and quality. Valiela [55] found however, that when low quality food is of high abundance this is favourable for a consumer species which display feeding selectivity.

Our results demonstrate that gastropods that feed on a mixed algal diet does not exhibit higher growth rates compared to a single algal diet. Raubenheimer and Simpson [56] suggested that the advantage of a mixed diet is only possible if no single diet species approaches the consumer species’ optimal nutrient requirements [56] and when complementary food resources are available. Franzke [57] fed grasshoppers with natural mixtures of prey items and measured the fitness of the consumer species. They found that a mixed diet was optimal at some but not all sites, thus supporting the findings of Raubenheimer and Simpson [56]. We observed similar patterns. Snail fed a the best single algae species diet obtained the higest growth rate and shell length. Whether or not a mixed diet is beneficial is likely determined by the identity of the prey species.

## Conclusions

Our results demonstrate that a mixed-diet can support a higher growth rate of a consumer species than the average of single prey species diets. This might be explained by the discrepancy in algae cell size or compensatory feeding. The generalist herbivore did not obtain a higher growth rate when consuming a mixed-diet compared to the best single species diet. Moreover, it is not able to selectively feed and thereby obtain optimal growth by actively regulating their diet and consuming complementary prey. This is however possible for a selective grazer. This means that there is a higher probability of finding support for the balanced-diet hypothesis concerning selective grazers. Therefore, we would like to emphasize the importance of differentiating between selective and non-selective grazers when conducting diet mixing experiments.

## Supporting Information

S1 Table Somatic growth rates and shell heights. A table including the raw data of somatic growth rates and shell height of the snails used in the experiment.
(XLSX)
